# Variables Associated with Short-Term Weight Loss in a Cohort of Patients with Morbid Obesity According to Age and Three Types of Bariatric Surgery

**DOI:** 10.3390/jcm9113537

**Published:** 2020-11-02

**Authors:** Maria D. Alvarez-Bermudez, Flores Martin-Reyes, Luis Ocaña-Wilhelmi, Francisco J. Moreno-Ruiz, Juan Alcaide Torres, Diego Fernandez-Garcia, Sergio Valdes, Noelia Moreno-Morales, Eduardo Garcia-Fuentes, Francisco J. Tinahones, Lourdes Garrido-Sanchez

**Affiliations:** 1 CIBER Fisiopatología Obesidad y Nutrición (CIBERObn), Instituto Salud Carlos III, Km., 228220 Madrid, Spain; mar17alv@gmail.com (M.D.A.-B.); diegofernandezgarcia@hotmail.com (D.F.-G.); lourdes.garrido@ibima.eu (L.G.-S.); 2 Unidad de Gestión Clínica de Endocrinología y Nutrición, Hospital Universitario Virgen de la Victoria, 29010 Málaga, Spain; juan.alcaidetorres@gmail.com; 3 Instituto de Investigación Biomédica de Málaga-IBIMA, 29010 Málaga, Spain; floresmarey@hotmail.com (F.M.-R.); sergio.valdes@hotmail.es (S.V.); 4 Unidad de Gestión Clínica de Aparato Digestivo, Hospital Universitario Virgen de la Victoria, 29010 Málaga, Spain; 5 Departamento de Especialidades Quirúrgicas, Bioquímica e Inmunología, Universidad de Málaga, 29010 Málaga, Spain; luisowilhelmi@hotmail.com (L.O.-W.); javier.morenoruiz@gmail.com (F.J.M.-R.); 6 Unidad de Gestión Clínica de Cirugía General, Digestiva y Trasplantes, Hospital Universitario Virgen de la Victoria, 29010 Malaga, Spain; 7 Unidad de Gestión Clínica de Cirugía General, Digestiva y Trasplantes, Hospital Regional Universitario, 29010 Málaga, Spain; 8 CIBER Diabetes y Enfermedades Metabolicas Asociadas (CIBERDEM), Instituto Salud Carlos III, Km. 228220, Madrid, Spain; 9 Unidad de Gestión Clínica de Endocrinología y Nutrición, Hospital Regional Universitario, 29010 Málaga, Spain; 10 Departamento Fisioterapia. Facultad Ciencias de la Salud, Universidad de Malaga, 29010 Malaga, Spain; nmm@uma.es; 11 Departamento de Medicina y Dermatología, Universidad de Málaga, 29010 Málaga, Spain

**Keywords:** morbid obesity, bariatric surgery, excess weight loss

## Abstract

**Background** The percentage of excess weight lost (%EWL) after bariatric surgery (BS) shows great discrepancies from one individual to another. **Objective** To evaluate the %EWL one year after BS and to determine the existence of baseline biomarkers associated with weight loss. **Methods** We studied 329 patients with morbid obesity undergoing three types of BS (biliopancreatic diversion (BPD), Roux-en-Y gastric bypass (RYGB) and sleeve gastrectomy (SG)), depending on the %EWL one year after surgery: good responders (GR) (%EWL ≥ 50%) and non-responders (NR) (%EWL < 50%). **Results** The GR presented a higher percentage of change in anthropometric and biochemical variables compared to the NR group, even within each type of BS. There was a greater percentage of GR among those who underwent RYGB. The patients who underwent SG showed the lowest decrease in biochemical variables, both in GR and NR. Within the GR group, those with a lower age showed greater improvement compared to the other age groups. A %EWL ≥50% was negatively associated with the age and atherogenic index of plasma (AIP), and positively with the type of BS (RYGB). **Conclusions** The GR group was associated with lower age and AIP and undergoing RYGB. Additionally, those patients who underwent SG showed a lower metabolic improvement.

## 1. Introduction

Obesity and related comorbidities are health problems worldwide. In 2016, about 13% of the world’s adult population was shown to be obese [1]. Bariatric surgery (BS) is a therapeutic approach to obesity and its comorbidities, and results in huge benefits in comparison with pharmacological actions [2]. It has been demonstrated that weight loss due to surgery was greater than other conservative therapy effects, and produced better glucose control than medical therapy did [3]. That reduction reaches its maximum between 6 months and 3 years post-surgery [2,3,4].

Several studies suggest that weight loss is an important contributor to the health outcomes associated with BS [5]. It was considered favourable to lose at least 50% of excess weight after surgery [6]. However, the weight reduction after BS shows great discrepancies from one individual to another [7], with a minority of patients (5–20%) who do not achieve successful long-term weight loss [7]. Considering post-operative weight loss and subsequent recovery, a classification that stratifies the patients into good responders and non-responders was established [8].

Several studies have compared the percentage of excess weight loss (%EWL) between different types of BS [9] or even different techniques of the same type of surgery [10], while others have analyzed several preoperative predictors with discordant results [11,12]. However, the surgery response, even performed with the same technique, is variable among different subjects. This fact could be explained due to metabolic differences before undergoing BS which can be reflected in anthropometric measurements or baseline serum markers [13]. Therefore, these biomarkers may be able to predict the weight loss response rate. However, it is unclear which factors are associated with the amount of excess weight loss after BS [14,15]. A more profound study of pre-surgical factors that are able to predict treatment success would be very useful in clinical practice in order to select the best candidates for each intervention. Some preoperative factors are a predictor of weight loss after Roux-en-Y gastric bypass (RYGB), such as body mass index (BMI) and waist circumference, and age [16]. There is evidence that increased age is associated with a lower %EWL [17]. However, other studies suggest that for patients older than 50 and 60 years, age does not influence the outcome after BS [18].

According to this background, the aim of this retrospective observational study was the evaluation of the response regarding weight loss in the short term (1 year after BS) on all patients with morbid obesity who underwent different BS techniques, as well as to determine the existence of baseline biomarker associated to weight loss.

## 2. Experimental Section

### 2.1. Subjects of Study

Patients with morbid obesity (BMI > 40 kg/m^2^) in our retrospective observational study were selected among the 582 subjects that underwent BS at the Virgen de la Victoria University Hospital and at the Regional University Hospital of Malaga between 2008–2017 and consented to participate in the study. From those, only those patients that attended at baseline and at the follow-up one year after BS, and with data in all the variables analyzed were included (*n* = 329). There are no significant differences in biochemical and anthropometric variables and comorbidities between patients included and not included in this study (data not shown). Three types of surgical techniques (sleeve gastrectomy (SG), biliopancreatic diversion (BPD) and RYGB) were performed. The surgical technique used depended on the multidisciplinary team that followed the patient. RYGB and SG were performed at the Regional University Hospital, and BPD and SG were performed at the Virgen de la Victoria University Hospital. The characteristics of these techniques have been shown in previous studies [19,20]. RYGB consists of a small longitudinal gastric pouch (20 mL) created along the lesser curvature that is totally separated from the main stomach. The jejunum is divided 40 cm distal to the ligament of Treitz and advanced in an antecolic/antegastric position to create a 125 cm Roux-en-Y limb, which is anastomosed to the gastric pouch [20]. BPD consists of a distal gastrectomy with a long Roux-en-Y reconstruction with the enteroenteric anastomosis performed 50 cm proximal to the ileocecal valve and the gastroenteric anastomosis performed 250 cm proximal to the ileocecal valve, with 200 mL of gastric volume [19]. SG is a technique that involves a longitudinal section parallel to the gastric lesser curvature supervised by the Fouche probe. The vascularization of the stomach is not compromised as the arterial supply of the celiac trunk remains intact [19]. The SG technique was similar in these two hospitals. The study was conducted in accordance with the guidelines laid down in the Declaration of Helsinki. All participants gave their informed consent to participate in this study prior to BS, and the study was reviewed and approved by the Ethics Committee of the Provincial Research of Malaga. The patients with morbid obesity were evaluated at baseline (prior to BS) and were followed up one year after the intervention by a multidisciplinary team (surgeons, endocrinologists and researchers), during which different anthropometric and biochemical data were prospectively collected. When the patients gave their consent, they were also informed that the data to be collected would be used for studies other than this one.

### 2.2. Clinical and Anthropometric Variables

Data were prospectively collected prior to BS and at 12 months in the postsurgical period. Anthropometric variables, both before BS and one year after, were measured in all the patients with morbid obesity included in the study. These included measurements of weight, height, waist and hip circumferences, and blood pressure. BMI was calculated as weight in kilograms divided by height in square metres. The %EWL was based on the excess weight compared to the weight corresponding to a BMI of 25 kg/m^2^ for each patient.

### 2.3. Assessment of Weight Change

We assessed %EWL as 100 × (preoperative weight—weight at the time of evaluation)/(preoperative weight—weight corresponding to BMI = 25 Kg/m^2^) [21]. The different patterns of weight loss were defined based on the EWL Reinhold criteria. Weight loss was considered insufficient when %EWL <50% in analogy with the Reinhold criteria [22]. The Reinhold criteria were modified by Christou et al. [23]. The patients with %EWL >50% of the weight at the beginning and throughout follow-up were considered as good responders (GR). On the other hand, patients with %EWL <50% of the weight at the beginning and up to the follow-up were considered as non-responders (NR).

### 2.4. Biochemical Variables

Blood samples were collected after a 12-h fast. The serum was separated and immediately frozen at −80 °C [19]. Serum glucose, cholesterol, triglycerides and HDL were analysed using an Advia Chemistry XPT autoanalyzer (Siemens Healthcare Diagnostics). Coefficients of variation for glucose, cholesterol, triglycerides and HDL were 1.8%, 2.5%, 3.9% and 4.5% respectively. The LDL was calculated from the Friedewald equation. Serum insulin levels were measured by immunoassay using an ADVIA Centaur autoanalyzer (Siemens Healthcare Diagnostics). Insulin resistance was calculated by the following formula: HOMA-IR = fasting insulin (μIU/mL) × fasting glucose (mmol/L)/22.5. The determination of leptin and adiponectin was performed by commercial enzyme-linked immunosorbent assay (ELISA) (Mediagnost, Germany, BLK Diagnostics, Spain, respectively). C-reactive protein (CRP) was performed by commercial ELISA (DRG Instruments GmbH, Marburg, Germany). Atherogenic index of plasma (AIP) was calculated as log (triglycerides/HDL) [24]. Total cholesterol/HDL cholesterol (TC/HDL) and triglycerides/HDL cholesterol (TG/HDL) index were also calculated [25]. The percentages of change (Δ) of the different anthropometric and biochemical variables at one year after BS were calculated using the following formula: (baseline variable—one-year variable) × 100/baseline variable [19].

Alterations in hydrocarbon metabolism were defined according to the criteria proposed by the American Diabetes Association (ADA) [26]. Type 2 diabetes mellitus (T2DM) was defined as two fasting plasma glucose values >125 mg/dl or glycated haemoglobin ≥6.5% or treatment with non-insulin hypoglycaemic agents or insulin. Criteria for hypertension diagnosis were current treatment with antihypertensive agents and/or systolic blood pressure >140 mmHg and/or diastolic blood pressure >90 mmHg [27]. Hypercholesterolemia was defined as total cholesterol >200 mg/dl or the use of cholesterol-lowering drugs [28].

### 2.5. Statistical Analysis

The statistical analysis was performed with SPSS (Windows 15.0; SPSS, Chicago, IL, USA). Data were expressed as mean ± standard deviation for continuous variables and as percentages for categorical variables. Student’s *t*-test was performed to assess differences between two means. The differences in the variables within the same group, before and after BS, were compared with the Student’s t-test for paired samples. Comparison between the results of the different groups was made with the one-way ANOVA and the post hoc analysis with the Bonferroni test. A Chi-square test was used to evaluate the degree of association between categorical variables. Pearson correlation coefficients were calculated to estimate the lineal associations between variables. The strength of association between variables was analysed by multivariate logistic regression models controlled for potential confounders such as age, sex, BMI at baseline, among others. Values were considered to be statistically significant when *p* ≤ 0.05.

## 3. Results

We followed 329 patients with morbid obesity during the first year after BS. Table 1 shows the characteristic of patients included in this study according to the type of BS. Patients underwent BPD were slightly more obese (higher weight (*p* = 0.001), BMI (*p* = 0.001), and waist (*p* = 0.045) and hip circumferences (*p* = 0.018)), and those underwent SG had lower glucose levels (*p* = 0.004). Patients lost the same total weight regardless of the type of BS (BPD: 44.2 ± 15.5 kg; RYGB: 48.4 ± 16.8 kg; SG: 44.8 ± 17.7 kg; *p* = 0.230). However, there was a lower percentage of total weight-loss (Δ-Weight) after BPD (29.7 ± 8.9%) than after RYGB (35.2 ± 8.1%) and SG (32.6 ± 9.3%) (*p* = 0.002).

The characteristics of patients classified according to the %EWL are presented in Table 2. Those patients with %EWL <50% presented a higher age (*p* = 0.031), baseline BMI (*p* = 0.005) and hip circumference (*p* = 0.037) than those with %EWL ≥50%. There was a decrease in the percentage of comorbidities after BS, both in the group of patients with %EWL <50% and with %EWL ≥50% (Table 2). However, these changes were more significant in those with %EWL ≥50%.

### 3.1. Association Between %EWL and the Variables Studied

The next step was to analyse the linear association between %EWL and anthropometric and biochemical variables through correlation analysis. There was a significant linear association between %EWL and age (*r* = −0.302, *p* < 0.001), weight (*r* = 0.280, *p* < 0.001), BMI (*r* = 0.259, *p* < 0.001), waist (*r* = 0.215, *p* < 0.001) and hip circumference (*r* = 0.287, *p* < 0.001) and AIP (*r* = −0.211, *p* < 0.001). No other significant associations were found (data not shown).

The variables associated with a %EWL ≥50% in a logistic regression model were the age, AIP and the type of BS (RYGB) (Table 3). This regression was adjusted for sex, BMI, HOMA-IR, CRP and hypertension (yes/no), which are related to the metabolic alterations most frequently associated with the presence and development of obesity: insulin resistance (HOMA-IR) [29], inflammation (CRP) [30] and hypertension [31].

### 3.2. %EWL According to the Type of BS

One year after BS, 80.5% of patients reached %EWL ≥50%. When analysed according to the type of BS, 86.7% of patients who underwent RYGB were GR. For SG, 82.2% were GR and of those undergoing BPD, 68.2% were GR. There was a greater percentage of patients who underwent RYGB who reached %EWL ≥50% compared to the other types of BS (*p* = 0.012).

Table 4 shows the characteristics of patients classified according to %EWL and the surgical technique used. A worse metabolic profile was found in those patients with %EWL <50%, both within BPD and SG groups. Within RYGB, we did not find significant differences.

### 3.3. %EWL and Comorbidities According the Type of BS

We analysed whether there were significant differences in these comorbidities within each type of BS, according to %EWL (Table 4). Within the SG group, we found a higher percentage of patients with T2DM (*p* = 0.034) and hypertension (*p* = 0.017) in the group of patients with %EWL <50%. No significant differences were observed within the BPD and RYGB groups.

### 3.4. %EWL According to Sex

Regarding sex, there were no significant differences in the percentage of women and men between the group of patients with %EWL ≥50% or with %EWL <50%.

### 3.5. %EWL According to Age

When the age was classified in quartiles (≤37 years, >37 and ≤44 (37–44) years, >44 and ≤52 (45–52) years and >52 years), we did not find significant differences in the percentage of GR, although a tendency was observed: the age ≤37 years group: 85.5%; the 37–44 years group: 82.7%; the 45–52 years group: 78.7%; the >52 years group: 75.3%.

Subsequently, we analysed whether there were significant differences according to the %EWL within each age group (Table 5). The main differences were within the 37–44 years group, with higher levels in the group of patients with %EWL <50%.

### 3.6. %EWL and Comorbidities According to Age

We also analysed whether there were significant differences in the comorbidities within each age group according to %EWL (Table 5). No significant differences were observed in the group of patients age ≤37 years and 45–52 years. Within the 37–44 years group, we observed a higher percentage of patients with T2DM (*p* = 0.035) and hypercholesterolemia (*p* = 0.048) in the group of patients with %EWL <50%. Finally, within the >52 years group, we observed a higher percentage of hypertensive patients (*p* = 0.049) in the group of patients with %EWL <50%.

### 3.7. %EWL and Percentage Change (Δ) of Anthropometric and Biochemical Variables

We analysed the Δ-anthropometric and Δ-biochemical variables according to the %EWL. Those patients with %EWL ≥ 50% presented a significant higher percentage of change compared to the group with %EWL < 50% in Δ-weight (*p* < 0.001), Δ-IMC (*p* < 0.001), Δ-waist (*p* < 0.001), Δ-hip (*p* < 0.001), Δ-triglycerides (*p* = 0.002), Δ-leptin (*p* < 0.001), Δ-HOMA-IR (*p* < 0.001), Δ-adiponectin (*p* = 0.024) and Δ-TG/HDL index (*p* = 0.009). No other significant differences were found.

### 3.8. %EWL and Δ-Anthropometric and Δ-Biochemical Variables According to Type of BS

We also analysed the Δ-anthropometric and Δ-biochemical variables according to %EWL and type of BS (Table 6). Higher Δ-anthropometric and Δ-biochemical variables were found in those patients with %EWL ≥50% within the three types of BS.

Within the %EWL <50% group (Table 6), the patients underwent SG showed the lowest decrease in glucose (*p* = 0.035) and CRP levels (*p* = 0.015), and the greatest increase in cholesterol (*p* < 0.001), HDL (*p* = 0.017) and LDL (*p* < 0.001) levels. The patients underwent RYGB showed the lowest decrease in waist circumference (*p* = 0.019).

Within the %EWL ≥50% group (Table 6), the patients underwent SG showed the lowest decrease in glucose (*p* = 0.023) and TC/HDL (*p* = 0.006) levels, and the greatest increase in cholesterol (*p* < 0.001), HDL (*p* < 0.001), LDL (*p* < 0.001) and adiponectin (*p* = 0.004) levels. The patients underwent BPD showed the lowest decrease in weight (*p* = 0.042), BMI (*p* = 0.042), waist circumference (*p* = 0.045), triglycerides (*p* < 0.001), TG/HDL (*p* < 0.001) and AIP (*p* = 0.038).

### 3.9. %EWL and Δ-Anthropometric and Δ-Biochemical Variables According to Age

We also analysed the Δ-anthropometric and Δ-biochemical variables according to %EWL and age (Table 7). Higher Δ-anthropometric and Δ-biochemical variables were found in those patients with %EWL ≥50% within the four groups of age.

Within the %EWL <50% group (Table 7), there was no age group that was clearly different compared to the other groups.

Within the %EWL ≥50% group (Table 7), the ≤37 years group showed the greatest decreases compared to the other groups (in weight (*p* < 0.001), BMI (p < 0.001), waist (*p* = 0.001) and hip circumference (*p* = 0.002), systolic (*p* = 0.012) and diastolic blood pressure (*p* = 0.025) and HOMA-IR (*p* = 0.035)). The >52 years group showed the greatest decrease in glucose (*p* = 0.016). The 45–52 years group showed the greatest increase in adiponectin (*p* = 0.014).

## 4. Discussion

The main finding of our study is that the main variables associated with a higher chance of a good weight loss response were age and the type of BS (RYGB), with the weight loss and AIP improvement being associated with each other; those patients with less age (≤37 years) are those that show a greater improvement in the variables analysed, mainly in the group with %EWL ≥50%. Additionally, those patients who underwent SG were those who showed a lower metabolic improvement (triglycerides, insulin, HOMA-IR, leptin and CRP), mainly in the group with %EWL <50%.

We found that BS achieves successful results in most of the variables studied in the short term, with an adequate percentage of post-surgery success 12 months after BS. Moreover, we observed significant improvements both in the non-responders group and in the good responder group. However, there are slight differences. The good responder group presents a greater improvement, and not only in anthropometric variables. However, the effects of all types of BS are not equal [32]. We also found slight differences between the effects of three types of BS. RYGP produced a higher %EWL than SG and, mainly, BPD. However, these patients had worse anthropometric characteristics. This could be conditioning that the %EWL was slightly lower in these patients. Other variables not considered in this study, such as the metabolic state of adipose tissue, could affect %EWL. The worse anthropometric characteristics of patients underwent BPD could alter the metabolism of adipose tissue: higher adipocyte hypertrophy is closely associated with a metabolic dysregulation [33], which could be associated with the evolution of these patients after BS [34]. On the other hand, and according to our results, there are studies showing a similar %EWL with RYGB [13,23]. We found that the percentage of patients with morbid obesity who do not achieve the desired weight loss depends on the type of surgery. Ma et al. [17] found that 85% of the patients who underwent gastric bypass achieved ≥50% EWL [17]. With regard to patients undergoing SG, and according to our results, other studies showed %EWL between 43 to 86% [35]. However, SG was the type of BS that produced the least improvement in the metabolic profile, mainly in the group with %EWL < 50%. This agrees with previous studies in which techniques with an important malabsorptive component were more effective than SG for weight outcomes and improvement of obesity-related comorbidities [36].

In addition to the effect of the type of bariatric surgery on %EWL, we observed that the weight loss is influenced by the age of the patient. Our study shows a tendency, with a higher %EWL in younger patients, as in other studies [16,17,18]. Different studies suggested that patients older than 50 years lost 40% less weight 2 years after BS than younger patients [18,37], with morbidity and mortality rates higher in older patients [11,38]. In addition to the influence of age on %EWL, we also found that there are differences between good responders and non-responders within each group of age. In general, good responder patients have a better baseline biochemical and anthropometric profile than those non-responders, mainly in the 37–44 years group. However, this better profile disappears as age increases. We observed that patients with a lower age showed some predictive factors of a %EWL ≥50%, mainly the group with 37–44 years; a better anthropometric (weight, BMI, waist and hip circumference), glycaemic (glucose, insulin and HOMA-IR) and atherosclerotic (triglycerides, TG/HDL index and AIP) profile. Overall, these patients have a better metabolic profile. These results suggest that there is a group of young patients with morbid obesity with a better metabolic profile, who are more favourable to adequate weight loss.

The last variable that we found to be associated with %EWL is AIP. It is a strong risk factor for atherosclerosis and a predictive factor for emergency cardiovascular events [39]. This index significantly improved after BS in all patients. Additionally, baseline AIP is lower in the group of BPD with %EWL ≥50%. As is known, and as we also found, BPD produces a significant improvement of the lipidic profile, which is closely linked to cardiovascular risk. Immediate post-surgical results showed a greater improvement in the lipid profile in patients who underwent BPD than in those who underwent SG [40]. 

We also found a significant decrease in the percentage of patients with T2DM, hypertension and hypercholesterolemia after BS regardless of %EWL (Table 2). In addition to weight loss, other metabolic factors related to surgical technique may determine the evolution of these comorbidities. There are numerous studies that support the results obtained in our study regarding weight reduction and control of cardiovascular risk factors in the short term. Piché et al. showed a reduction in comorbidities such as hypertension, T2DM and dyslipidemia [41]. Other studies found similar results in patients who underwent RYGB and SG after a 17-month follow-up [42]. Although the comorbidities decrease after BS, weight loss (%EWL <50% or ≥50%) in those patients undergoing BPD and RYGB was not associated with a higher or lower pre-surgical presence of T2DM, hypertension and hypercholesterolemia. However, a lower presence of T2DM and hypertension in those patients undergoing SG was associated with a %EWL ≥50%. This suggests that baseline characteristics of patients may be associated with weight loss [4].

The present study is not exempt from limitations. We only analysed a few variables that can potentially influence the results that are measured. Additionally, although potential post-surgery features that could be determinants of the final effects were not considered, it is a strength that all patients followed homogeneous therapeutic recommendations after each type of BS. We also used 50% as a cut-off for the EWL, although other cut-offs could have been used. Studies for short- and long-term weight-loss show different results. While some reviews show similar %EWL with SG and RYGB at short and long-term [43], other reviews show better results with RYGB [44], with a greater treatment failure after six years for SG [44]. Most of the studies demonstrated and maintained weight loss through follow-up at five years and even for longer intervals (up to 11 years) [43]. However, a slow weight gain between the second and third years of postsurgery follow-up is found, increasing up to 5 years postsurgery [45]. Although, based on previous reviews, our results could be generalized to long-term weight loss, we cannot confirm this hypothesis without data. A long-term follow-up time would be necessary to compare the different surgical techniques and to determine the true variables associated with weight loss after BS, because many patients recover weight, as well as the associated comorbidities after the first years [3,4]. Another limitation that should be mentioned is that two hospitals contributed patients and shared only one surgery. However, this is performed with the same technical characteristics, so it would hardly affect the results obtained.

In conclusion, we show that the relevant variables associated with a %EWL ≥50% after 12 months of follow-up after BS were the type of surgery, mainly RYGB, and age, which is also associated with AIP. Our study confirms that BS, and mainly RYGB, is an effective procedure to metabolically the patients with morbid obesity, even in those non-responders. SG seems to be the one that showed a lower metabolic improvement, mainly in the non-responders group. More extensive knowledge would serve to predict the response to surgery.

## Figures and Tables

**Table 1:** Characteristics of patients with morbid obesity at baseline classified according to the type of bariatric surgery.

	Pre-Surgery		
	BPD	RYGB	SG
N (%)	66	83	180
Sex (men/women)	24/42	20/63	59/121
Age (years)	42.9 ± 9.6	43.9 ± 9.4	44.1 ± 10.1
Weight (Kg)	147.4 ± 22.8 ^a^	135.3 ± 23.7 ^b^	135.1 ± 23.7 ^b^
BMI (kg/m^2^)	53.8 ± 6.5 ^a^	49.7 ± 8.0 ^b^	50.2 ± 7.7 ^b^
Waist (cm)	141.4 ± 16.7 ^a^	135.8 ± 15.4 ^b^	134.4 ± 13.4 ^b^
Hip (cm)	152.6 ± 13.3 ^a^	147.6 ± 17.2 ^b^	146.1 ± 14.9 ^b^
SBP (mmHg)	138.5 ± 20.9	137.9 ± 18.3	137.5 ± 19.1
DBP (mmHg)	84.9 ± 14.5	81.7 ± 12.5	83.0 ± 10.4
Glucose (mg/dL)	117.1 ± 40.6 ^a^	119.8 ± 51.9 ^a^	104.8 ± 49.9 ^b^
Total cholesterol (mg/dL)	193.3 ± 44.9	197.8 ± 37.0	187.5 ± 33.7
Triglycerides (mg/dL) *	151.3 (101.2–208.0)	127.0 (99.0–184.0)	122.0 (77.1–145.5)
HDL (mg/dL)	44.6 ± 10.4	47.1 ± 11.5	44.4 ± 11.3
LDL (mg/dL)	119.9 ± 36.3	121.6 ± 31.6	109.8 ± 31.7
Insulin (μIU/mL) *	19.2 (16.2–24.3)	15.3 (13.2–21.9)	13.1 (11.1–20.4)
HOMA-IR *	5.03 (4.2–7.7)	4.01 (3.3–6.2)	3.6 (2.6–4.9)
Leptin (ng/mL) *	58.8 (52.3–84.6)	62.3 (49.0–81.5)	41.9 (33.4–57.4)
Adiponectin (µg/mL)	8.8 ± 4.5	7.9 ± 3.8	8.2 ± 3.5
CRP (mg/L) *	5.3 (3.9–9.3)	9.7 (7.8–16.4)	3.3 (1.5–8.1)
TG/HDL *	3.7 (1.9–5.6)	2.7 (2.1–3.5)	2.1 (1.7–3.2)
TC/HDL	4.3 ± 1.2	4.3 ± 1.1	4.3 ± 1.2
AIP	0.46 ± 0.26	0.45 ± 0.27	0.47 ± 0.27
Comorbilities			
%Patients with T2DM (n)	39.4 (26)	44.6 (37)	32.7 (59)
%Patients with hypertension (n)	74.3 (49)	82.0 (68)	72.7 (131)
%Patients with hypercholesterolemia (n)	47.0 (31)	67.5 (56)	41.7 (75)

The results are given as the mean ± SD. * These results are given as median (interquartile range). BMI: Body mass index. SBP: Systolic blood pressure; DBP: Diastolic blood pressure; HOMA-IR: homeostasis model assessment of insulin resistance index. CRP: C-reactive protein. TG: triglycerides; TC: total cholesterol. AIP: atherogenic index of plasma. Different letters show significant differences between the means of the three types of bariatric surgery: *p* < 0.05.

**Table 2:** Characteristics of patients with morbid obesity at baseline and one year after bariatric surgery, classified according to the percentage of excess weight loss (%EWL).

	Pre-Surgery		Post-Surgery	
	%EWL <50%	%EWL ≥50%	%EWL <50%	%EWL ≥50%
N (%)	64 (19.4%)	265 (80.6%)	64 (19.4%)	265 (80.6%)
Sex (men/women)	18/46	85/180		
Age (years)	46.6 ± 10.2	43.9 ±9.7 ^1^		
Weight (Kg)	142.2 ± 24.9	137.1 ± 23.7	111.9 ± 17.6 ^‡^	87.9 ± 14.1 ^2,‡^
BMI (kg/m^2^)	53.4 ± 8.6	50.1 ± 7.4 ^1^	42.0 ± 5.8 ^‡^	32.1 ± 4.5 ^2,‡^
Waist (cm)	140.9 ± 14.3	136.6 ± 15.8	120.0 ± 12.5 ^‡^	102.4 ± 12.2 ^2,‡^
Hip (cm)	152.5 ± 14.5	147.1 ± 15.4 ^1^	132.1 ± 12.1 ^‡^	113.5 ± 12.8 ^2,‡^
SBP (mmHg)	142.8 ± 19.9	137.8 ± 18.6	131.6 ± 19.6 ^‡^	127.9 ± 20.4 ^‡^
DBP (mmHg)	84.5 ± 10.7	82.7 ± 11.9	81.4 ± 13.0 ^‡^	76.7 ± 12.3 ^1,‡^
Glucose (mg/dL)	115.3 ± 34.1	110.7 ± 41.9	91.6 ± 12.2 ^‡^	83.9 ± 15.5 ^2,‡^
Total cholesterol (mg/dL)	196.7 ± 39.1	188.1 ± 36.7	182.3 ± 50.6	173.7 ± 38.2 ^‡^
Triglycerides (mg/dL) *	183.0 (123.2–234.5)	122.1 (92.9–168.0)	147.0 (106.5–168.0) ^†^	82.0 (60.5–114.0) ^2,‡^
HDL (mg/dL)	44.9 ± 10.9	45.5 ± 11.3	50.7 ± 15.5 ^†^	53.7 ± 13.3 ^‡^
LDL (mg/dL)	120.8 ± 34.0	113.9 ± 32.2	108.8 ± 40.0	103.5 ± 32.8 ^‡^
Insulin (μIU/mL) *	15.3 (13.4–20.6)	17.1 (12.9–23.6)	9.1 (7.4–11.4) ^‡^	7.5 (5.2–9.1) ^2,‡^
HOMA-IR *	4.3 (3.5–5.5)	4.4 (3.3–6.7)	2.1 (1.5–2.4) ^‡^	1.5 (1.1–1.9) ^2,‡^
Leptin (ng/mL) *	78.4 (45.8–88.1)	58.2 (43.7–75.0)	25.4 (15.1–39.2) ^‡^	12.0 (8.8–16.0) ^2,‡^
Adiponectin (µg/mL)	9.1 ± 4.0	7.9 ± 4.1	11.1 ± 5.3	13.8 ± 6.7 ^‡^
CRP (mg/L) *	7.5 (5.7–11.7)	8.1 (3.9–11.4)	0.3 (0.2–1.7) ^†^	0.7 (0.3–2.3) ^‡^
TG/HDL *	4.0 (2.7–5.9)	2.6 (1.9–3.6)	3.1 (2.9–4.0) ^‡^	1.6 (1.0–2.4) ^2,‡^
TC/HDL	4.6 ± 1.2	4.3 ± 1.1	3.7 ± 1.1 ^‡^	3.3 ± 0.9 ^1,‡^
AIP	0.50 ± 0.26	0.45 ± 0.27	0.36 ± 0.25 ^‡^	0.19 ± 0.21 ^2,‡^
Comorbilities				
%Patients with T2DM (n)	45.3 (29)	35.4 (94)	3.2 (2) ^†^	0.8 (2) ^‡^
%Patients with hypertension (n)	82.8 (53)	73.5 (195)	60.9 (39) ^†^	49.1 (130) ^‡^
%Patients with hypercholesterolemia (n)	56.2 (36)	48.3 (128)	39.0 (25) ^†^	28.3 (75) ^‡^

The results are given as the mean ± SD. * These results are given as median (interquartile range). BMI: Body mass index. SBP: Systolic blood pressure; DBP: Diastolic blood pressure; HOMA-IR: homeostasis model assessment of insulin resistance index. CRP: C-reactive protein. TG: triglycerides; TC: total cholesterol. AIP: atherogenic index of plasma. Significant differences between patients with morbid obesity with %EWL < 50 and those with %EWL ≥50%, both baseline and one year after bariatric surgery: ^1^ *p* < 0.05; ^2^ *p* < 0.001. Significant differences in patients with morbid obesity between before and after bariatric surgery, both in those with %EWL <50% and in those with %EWL ≥ 50%: ^†^ *p* < 0.05; ^‡^ *p* < 0.001.

**Table 3:** Variables associated with a %EWL ≥50% (%EWL <50% (0) and %EWL ≥50% (1)) obtained from a logistic regression model.

	B Coefficient	*p*	OR	(95% Confidence Interval)
Sex (women = 0/men = 1)	−0.462	0.449	0.630	0.191–2.081
Age	−0.066	0.050	0.936	0.876–0.999
BMI	−0.001	0.977	0.999	0.913–1.092
Hypertension (yes = 0/no = 1)	−0.673	0.416	0.510	0.101–2.579
AIP	−3.188	0.019	0.041	0.003–0.591
Type of surgery		0.045		
Type of surgery (SG)	0.958	0.196	2.607	0.610–11.147
Type of surgery (RYGB)	1.850	0.014	6.360	1.465–27.606
HOMA-IR	0.106	0.227	1.112	0.936–1.320
CRP	−0.015	0.692	0.985	0.914–1.062

**Table 4:** Baseline characteristics of patients with morbid obesity classified according to %EWL and the type of bariatric surgery.

	%EWL <50%			%EWL ≥50%		
	BPD	RYGB	SG	BPD	RYGB	SG
N (% within each type of BS)	21 (31.8%)	11 (13.3%)	32 (17.8%)	45 (68.2%)	72 (86.7%)	148 (82.2%)
Sex (men/women)	8/13	2/9	8/24	16/29	18/54	51/97
Age (years)	42.0 ± 8.9	46.6 ± 11.8	48.1 ± 9.9	41.6 ± 10.1	43.5 ± 9.1	44.4 ± 9.9
Weight (Kg)	148.9 ± 24.6	137.8 ± 23.5	137.5 ± 26.6	146.6 ± 21.6 ^a^	134.8 ± 23.1 ^b^	134.7 ± 23.8 ^b^
BMI (kg/m2)	54.4 ± 7.8	52.4 ± 8.2	52.3 ± 9.8	53.5 ± 5.9 ^a^	49.9 ± 7.6 ^b^	49.2 ± 7.4 ^b^
Waist (cm)	143.8 ± 14.7	135.0 ± 13.2	139.5 ± 13.8	140.3 ± 15.8	137.1 ± 16.2	135.1 ± 15.6
Hip (cm)	153.6 ± 10.4	147.5 ± 20.3	150.6 ± 14.7	152.2 ± 13.3 ^a^	147.6 ± 16.9 ^a,b^	145.2 ± 14.8 ^b^
SBP (mmHg)	138.0 ± 23.5	134.6 ± 5.5	145.1 ± 17.4	138.7 ± 20.2	137.6 ± 18.2	136.7 ± 18.2 ^1^
DBP (mmHg)	84.4 ± 15.1	77.0 ± 3.4	85.7 ± 8.8	85.1 ± 14.9	82.1 ± 12.8	82.5 ± 10.8
Glucose (mg/dL)	120.2 ± 31.6	116.2 ± 52.9	105.7 ± 20.1	115.4 ± 44.9 ^a,b^	120.2 ± 52.2 ^a^	103.4 ± 32.9 ^b,1^
Total cholesterol (mg/dL)	200.0 ± 42.9	207.6 ± 45.8	183.4 ± 31.6	189.6 ± 46.1 ^a,b^	196.5 ± 35.9 ^a^	182.3 ± 34.2 ^b^
Triglycerides (mg/dL) *	165.5 (130.0–210.0)	175.0 (96.0–183.0)	179.0 (143.0–222.0)	105.0 (87.0–169.0) ^b,1^	138.5 (100.0–202.0) ^a^	110.0 (75.5–128.4) ^a,b^
HDL (mg/dL)	40.6 ± 10.4 ^b^	50.0 ± 9.3 ^a^	45.4 ± 11.7 ^a,b^	46.7 ± 9.8 ^1^	46.7 ± 11.8	44.2 ± 11.2
LDL (mg/dL)	125.7 ± 39.6	129.0 ± 40.7	109.3 ± 25.3	117.2 ± 34.5	118.3 ± 32.7	109.9 ± 33.0
Insulin (μIU/mL) *	17.9 (13.3–22.2)	17.0 (13.7–25.8)	12.5 (11.9–15.4)	22.2 (16.7–28.6)	16.9 (13.5–24.1)	17.3 (11.0–22.5)
HOMA-IR *	5.4 (4.2–6.4)	4.1 (3.4–9.9)	3.5 (2.9–4.3)	6.9 (4.2–9.2)	4.3 (3.3–6.8)	3.8 (2.7–5.5)
Leptin (ng/mL) *	88.1 (57.7–91.2)	78.7 (42.0–122.3)	39.8 (39.5–106.0)	56.8 (51.0–67.0) ^1^	63.2 (46.0–94.0)	43.8 (35.5–68.8)
Adiponectin (µg/mL)	8.6 ± 4.2	9.1 ± 4.4	9.8 ± 2.3	8.8 ± 4.7	7.1 ± 3.7	7.9 ± 3.6
CRP (mg/L) *	5.9 (3.4–9.4)	7.9 (2.4–15.6)	9.4 (4.8–10.8)	4.3 (3.0–9.8) ^a,b^	8.5 (3.8–12.7) ^a^	3.3 (1.4–9.2) ^b^
TG/HDL *	3.7 (2.9–5.8)	3.1 (2.5–3.5)	2.8 (2.5–5.0)	2.5 (1.7–3.9) ^2^	3.0 (2.0–4.0)	2.2 (1.5–3.1)
TC/HDL	5.2 ± 1.2 ^a^	4.2 ± 0.8 ^b^	4.2 ± 1.2 ^b^	4.1 ± 1.1 ^2^	4.4 ± 1.2	4.3 ± 1.2
AIP	0.60 ± 0.27	0.43 ± 0.19	0.47 ± 0.27	0.38 ± 0.23 ^2^	0.47 ± 0.28	0.47 ± 0.27
Comorbilities						
%Patients with T2DM (n)	42.9 (9)	36.7 (4)	46.8 (15)	37.8 (17)	45.8 (33)	29.1 (43) ^1^
%Patients with hypertension (n)	66.7 (14)	100.0 (11)	87.5 (28)	80.0 (36)	80.5 (58)	69.6 (103) ^1^
%Patients with hypercholesterolemia (n)	57.1 (12)	81.8 (9)	40.6 (13)	42.2 (19)	65.3 (47)	41.9 (62)

The results are given as the mean ± SD. * These results are given as median (interquartile range). BMI: Body mass index. SBP: Systolic blood pressure; DBP: Diastolic blood pressure; HOMA-IR: homeostasis model assessment of insulin resistance index. CRP: C-reactive protein. TG: triglycerides; TC: total cholesterol; AIP: atherogenic index of plasma. Different letters show significant differences between the means of the three types of bariatric surgery: *p* < 0.05. Significant differences between patients with morbid obesity with %EWL <50 and those with %EWL ≥50% according to the type of bariatric surgery: ^1^ *p* < 0.05; ^2^ *p* < 0.01 and ^3^ *p* < 0.001 (to simplify these data, significant differences are marked in bold).

**Table 5:** Baseline characteristics of patients with morbid obesity classified according to the percentage of excess weight loss (%EWL) and age.

	%EWL <50%				%EWL ≥50%			
	≤37 Years	37–44 Years	45–52 Years	>52 Years	≤37 Years	37–44 Years	45–52 Years	>52 Years
N (% within each group of age)	12 (14.5%)	14 (17.3%)	17 (21.3%)	21 (24.7%)	71 (85.5%)	67 (82.7%)	63 (78.7%)	64 (75.3%)
Sex (men/women)	4/8	5/9	5/12	4/17	23/48	16/51	23/40	23/41
Age (years)	31.2 ± 4.6 ^d^	40.6 ± 1.9 ^c^	48.7 ± 2.1 ^b^	57.9 ± 2.4 ^a^	31.4 ± 4.7 ^d^	41.4 ± 1.8 ^c^	47.8 ± 2.1 ^b^	56.4 ± 3.4 ^a,1^
Weight (Kg)	146.2 ± 28.1 ^a,b^	159.9 ± 24.5 ^a^	137.7 ± 20.9 ^b,c^	128.2 ± 21.4 ^c^	144.9 ± 24.9 ^a^	133.3 ± 21.0 ^b,2^	137.0 ± 22.8 ^b^	131.8 ± 23.2 ^b^
BMI (kg/m2)	52.6 ± 10.5 ^a,b^	58.6 ± 7.8 ^a^	51.2 ± 8.9 ^b^	51.3 ± 7.0 ^b^	51.8 ± 8.1 ^a^	49.7 ± 7.6 ^a,b,2^	49.4 ± 6.4 ^a,b^	49.1 ± 7.0 ^b^
Waist (cm)	148.6 ± 16.6 ^a^	146.4 ± 16.7 ^a,b^	134.7 ± 11.2 ^c^	136.9 ± 13.3 ^b,c^	137.3 ± 16.9 ^a,b^	132.6 ± 15.4 ^b,2^	136.9 ± 14.2 ^a,b^	139.1 ± 15.6 ^a^
Hip (cm)	152.9 ± 18.8	156.8 ± 15.4	149.3 ± 13.1	149.8 ± 14.9	151.0 ± 16.4 ^a^	146.2 ± 14.2 ^a,b,2^	144.7 ± 16.3 ^b^	145.7 ± 13.6 ^ab^
SBP (mmHg)	127.8 ± 9.8 ^b^	135.2 ± 12.4 ^b^	138.7 ± 22.2 ^b^	156.7 ± 15.8 ^a^	135.6 ± 15.5 ^b^	135.3 ± 18.2 ^b^	136.1 ± 20.6 ^b^	143.2 ± 20.1 ^a,1^
DBP (mmHg)	83.8 ± 8.9	83.5 ± 9.3	83.6 ± 13.8	86.6 ± 9.3	82.0 ± 11.2	83.8 ± 12.1	81.3 ± 12.0	83.8 ± 12.2
Glucose (mg/dL)	111.7 ± 40.6	127.0 ± 38.3	107.1 ± 25.6	114.3 ± 33.6	97.0 ± 31.0 ^c^	107.1 ± 31.3 ^b,c,2^	111.6 ± 46.6 ^b^	126.2 ± 50.2 ^a^
Total cholesterol (mg/dL)	189.7 ± 35.2	198.2 ± 45.3	185.1 ± 39.9	206.4 ± 36.5	184.7 ± 36.6	185.4 ± 37.2	190.2 ± 37.3	190.7 ± 36.5
Triglycerides (mg/dL) *	194.5 (127.5–351.4)	1710 (150.5–216.0)	149.9 (112.7–185.4)	179.0 (122.5–193.0)	117.5 (71.0–147.0) ^b^	120.0 (89.5–187.5) ^ab,1^	105.0 (88.0–157.0) ^b^	138.8 (121.0–209.0) ^a^
HDL (mg/dL)	40.1 ± 8.1 ^b^	43.0 ± 11.2 ^b^	40.9 ± 9.4 ^b^	52.8 ± 10.8 ^a^	43.6 ± 11.7	45.4 ± 12.1	45.9 ± 9.4 ^1^	46.7 ± 11.9 ^1^
LDL (mg/dL)	114.5 ± 23.9	119.1 ± 39.1	118.0 ± 37.1	125.0 ± 33.4	114.2 ± 28.0	114.3 ± 34.1	114.3 ± 35.0	112.2 ± 33.0
Insulin (μIU/mL) *	15.2 (12.1–23.4)	25.8 (20.3–33.6)	13.1 (11.9–16.3)	15.4 (13.1–18.8)	18.4 (12.8–29.4)	18.1 (12.8–24.5)	20.1 (15.1–24.0)	16.8 (13.1–23.5)
HOMA-IR *	5.6 (4.1–6.1)	9.4 (5.9–14.2)	4.0 (3.0–4.3)	3.9 (3.3–4.7)	4.1 (2.7–7.5)	4.7 (3.5–6.9) ^1^	5.1 (3.8–7.5)	4.3 (3.3–7.1)
Leptin (ng/mL) *	67.0 (37.1–98.7)	90.7 (83.6–93.5)	60.8 (34.0–91.2)	68.3 (39.6–109.0)	59.1 (49.0–83.3)	53.4 (41.0–85.4)	62.3 (35.9–82.9)	53.0 (38.0–64.2)
Adiponectin (µg/mL)	5.4 ± 2.6 ^b^	7.9 ± 5.8 ^a,b^	9.8 ± 2.4 ^a^	10.8 ± 3.2 ^a^	7.5 ± 3.7	8.5 ± 4.7	8.6 ± 3.8	8.9 ± 3.9
CRP (mg/L) *	7.8 (4.3–28.9)	8.3 (4.1–17.7)	4.9 (3.4–5.7)	8.5 (5.5–13.2)	8.3 (3.4–11.3)	4.2 (1.8–8.9)	7.3 (1.7–11.8)	7.3 (3.8–9.8)
TG/HDL *	4.6 (3.3–11.5)	4.2 (2.8–5.8)	3.0 (2.9–4.0)	2.8 (2.5–4.2)	2.6 (1.5–3.1)	2.6 (1.7–3.9) ^1^	2.2 (2.0–3.5)	3.1 (2.1–5.6)
TC/HDL	4.8 ± 1.3	4.8 ± 1.4	4.6 ± 1.1	4.1 ± 0.9	4.4 ± 1.3	4.4 ± 1.2	4.2 ± 1.0	4.2 ± 1.2
AIP	0.55 ± 0.29	0.59 ± 0.27	0.51 ± 0.24	0.41 ± 0.26	0.43 ± 0.26	0.41 ± 0.27 ^1^	0.45 ± 0.25	0.51 ± 0.29
Comorbidities								
%Patients with T2DM (n)	25.0 (3)	57.1 (8)	47.0 (8)	47.6 (10)	18.3 (13)	26.8 (18) ^1^	34.9 (22)	62.5 (40)
%Patients with hypertension (n)	58.3 (7)	64.2 (9)	82.4 (14)	100.0 (21)	59.1 (42)	74.6 (50)	77.8 (49)	84.3 (54) ^1^
%Patients with hypercholesterolemia (n)	41.7 (5)	71.4 (10)	35.3 (6)	71.4 (15)	35.2 (25)	43.2 (29) ^1^	50.8 (32)	68.8 (44)

The results are given as the mean ± SD or * as median (interquartile range). BMI: Body mass index. SBP: Systolic blood pressure; DBP: Diastolic blood pressure; HOMA-IR: homeostasis model assessment of insulin resistance index. CRP: C-reactive protein. TG: triglycerides; TC: total cholesterol. AIP: atherogenic index of plasma. Different letters show significant differences between the means of the four groups of age: *p* < 0.05. Significant differences between patients with morbid obesity with %EWL <50 and those with %EWL ≥50% according to age: ^1^ *p*< 0.05; ^2^ *p* < 0.01 and ^3^ *p* < 0.001 (to simplify these data, significant differences are marked in bold).

**Table 6:** Percentage change (Δ) of anthropometric and biochemical variables of patients with morbid obesity classified according to the percentage of excess weight loss (%EWL) and the type of bariatric surgery.

	%EWL <50%			%EWL ≥50%		
	BPD	RYGB	SG	BPD	RYGB	SG
Δ-Weight	20.9 ± 7.5	22.1 ± 7.3	20.0 ± 5.2	34.3 ± 5.8 ^b,3^	36.0 ± 6.6 ^a,3^	35.2 ± 7.6 ^a,b,3^
Δ-BMI	20.9 ± 7.5	22.1 ± 7.3	20.0 ± 5.2	34.3 ± 5.8 ^b,3^	36.0 ± 6.6 ^a,3^	35.2 ± 7.6 ^a,b,3^
Δ-Waist	17.7 ± 5.5 ^a^	12.6 ± 7.1 ^b^	14.2 ± 5.5 ^a,b^	22.4 ± 6.1 ^b,1^	25.4 ± 8.1 ^a,3^	24.9 ± 7.7 ^a,b,3^
Δ-Hip	14.2 ± 3.7	11.0 ± 9.2	12.6 ± 3.9	21.2 ± 5.6 ^3^	23.2 ± 14.1 ^3^	22.3 ± 6.6 ^3^
Δ-SBP	9.9 ± 10.5	7.1 ± 20.1	6.6 ± 12.0	9.7 ± 13.6	8.1 ± 13.8	6.2 ± 15.2
Δ-DBP	11.3 ± 12.9	2.6 ± 16.9	0.7 ± 16.0	8.4 ± 16.7	4.7 ± 22.5	5.1 ± 17.3
Δ-Glucose	20.5 ± 16.4 ^a^	22.4 ± 19.8 ^a^	9.0 ± 14.3 ^b^	20.7 ± 17.8 ^a,b^	24.6 ± 21.9 ^a^	14.6 ± 16.4 ^b^
Δ-Cholesterol	31.6 ± 14.8 ^a^	−0.5 ± 15.6 ^b^	−10.7 ± 24.2 ^b^	24.9 ± 18.0 ^a^	11.2 ± 22.6 ^b,1^	−6.0 ± 21.9 ^c^
Δ-Triglycerides *	24.9 (2.9–58.9)	18.9 (8.6–41.2)	5.46 (−10.9–26.2)	12.8 (−12.9–30.3) ^b^	34.6 (13.1–51.1) ^a^	41.0 (4.3–50.3) ^a,2^
Δ-HDL	0.8 ± 29.0 ^a^	−8.8 ± 25.1 ^a,b^	−30.5 ± 44.2 ^b^	−3.7 ± 26.5 ^a^	−20.2 ± 27.8 ^b^	−30.2 ± 31.1 ^b^
Δ-LDL	38.4 ± 14.0 ^a^	−5.5 ± 30.9 ^b^	−11.3 ± 36.2 ^b^	35.2 ± 21.2 ^a^	11.2 ± 34.2 ^a,b^	−2.3 ± 103.0 ^c^
Δ-Insulin *	47.4 (40.9–60.7)	20.1 (13.2–35.1)	16.3 (13.2–29.3)	62.9 (55.3–70.9) ^2^	54.6 (37.9–67.2)	58.4 (44.1–70.6) ^3^
Δ-HOMA-IR *	55.5 (46.2–77.6)	26.8 (21.6–39.8)	32.3 (29.1–42.4)	73.5 (64.1–76.8) ^1^	67.4 (52.3–77.7)	60.6 (51.7–77.2) ^3^
Δ-Leptin *	53.2 (47.8–69.6)	52.3 (48.5–61.2)	51.9 (50.8–56.5)	85.4 (77.9–88.3) ^2^	76.9 (70.7–85.1) ^2^	76.6 (64.0–79.9) ^1^
Δ-Adiponectin	−12.0 ± 37.0	−82.7 ± 59.5	−55.9 ± 60.1	−33.5 ± 56.0 ^a^	−119.1 ± 150.4 ^b^	−156.0 ± 124.3 ^b^
Δ-CRP *	95.8 (87.7–97.2) ^a^	71.7 (65.4–82.2) ^a,b^	65.2 (54.9–71.8) ^b^	84.8 (72.1–93.2)	83.8 (66.6–94.2)	89.2 (60.2–91.9) ^3^
Δ-TG/HDL *	31.4 (11.0–45.5)	35.7 (15.6–49.7)	8.8 (−17.4–34.6)	0.23 (−12.9–24.7) ^b^	47.5 (26.1–61.1) ^a^	52.6 (7.7–62.7) ^a,1^
Δ-TC/HDL	28.1 ± 17.9 ^a^	2.6 ± 29.7 ^b^	9.2 ± 25.6 ^b^	25.0 ± 16.4 ^a^	23.4 ± 19.8 ^a,1^	15.2 ± 22.0 ^b^
Δ-AIP	53.1 ± 154.0	19.9 ± 98.1	53.1 ± 77.6	21.2 ± 213.1 ^b^	49.6 ± 72.2 ^a,b^	70.0 ± 87.9 ^a,1^

The results are given as the mean ± SD or * as median (interquartile range). BMI: Body mass index. SBP: Systolic blood pressure; DBP: Diastolic blood pressure; HOMA-IR: homeostasis model assessment of insulin resistance index. CRP: C-reactive protein. TC: total cholesterol. TG: triglycerides; AIP: atherogenic index of plasma. Different letters show significant differences between the means of the three types of bariatric surgery: *p* < 0.05. Significant differences between patients with morbid obesity with %EWL <50 and those with %EWL ≥50% according to the type of bariatric surgery: ^1^ *p* < 0.05; ^2^ *p* < 0.01 and ^3^ *p* < 0.001 (to simplify these data, significant differences are marked in bold).

**Table 7:** Percentage change (Δ) of anthropometric and biochemical variables of patients with morbid obesity classified according to the percentage of excess weight loss (%EWL) and age.

	%EWL <50%				%EWL ≥50%			
	≤37 Years	37–44 Years	45–52 Years	>52 Years	≤37 Years	37–44 Years	45–52 Years	>52 Years
Δ-Weight	20.4 ± 6.3	23.5 ± 5.9	19.3 ± 7.1	19.5 ± 5.5	39.6 ± 7.3 ^a,3^	34.8 ± 6.6 ^b,3^	33.8 ± 6.0 ^b,3^	32.9 ± 5.8 ^b,3^
Δ-BMI	20.4 ± 6.3	23.5 ± 5.9	19.3 ± 7.1	19.5 ± 5.5	39.6 ± 7.3 ^a,3^	34.8 ± 6.6 ^b,3^	33.8 ± 6.0 ^b,3^	32.9 ± 5.8 ^b,3^
Δ-Waist	15.1 ± 5.2	15.8 ± 6.3	14.5 ± 5.1	14.8 ± 6.9	27.8 ± 6.8 ^a,3^	23.7 ± 9.1 ^b,2^	23.1 ± 5.2 ^b,3^	23.1 ± 7.5 ^b,3^
Δ-Hip	12.9 ± 4.7	13.7 ± 6.4	13.4 ± 4.0	11.8 ± 4.9	25.2 ± 6.7 ^a,3^	23.2 ± 10.3 ^a,b,3^	19.8 ± 10.8 ^c,3^	20.6 ± 5.6 ^b,c,3^
Δ-SBP	3.6 ± 9.8	4.9 ± 9.8	5.7 ± 16.2	13.1 ± 8.3	12.0 ± 12.2 ^a,1^	6.3 ± 14.4 ^b^	4.3 ± 15.1 ^b^	5.4 ± 15.9 ^b^
Δ-DBP	7.1 ± 14.4	0.5 ± 11.6	0.4 ± 21.0	6.4 ± 14.8	11.4 ± 15.6 ^a^	6.3 ± 17.8 ^a,b^	−0.6 ± 17.5 ^b^	3.8 ± 19.9 ^b^
Δ-Glucose	14.7 ± 19.1 ^a,b^	25.1 ± 16.5 ^a^	9.7 ± 17.5 ^b^	12.9 ± 12.2 ª^,b^	12.8 ± 19.1 ^c^	16.1 ± 15.4 ^b,c^	19.5 ± 17.5 ^a,b^	24.5 ± 18.7 ^a,1^
Δ-Cholesterol	18.2 ± 20.2 ^a^	11.3 ± 27.4 ^a,b^	−1.6 ± 32.7 ^a,b^	−3.1 ± 24.0 ^b^	9.4 ± 21.6	3.4 ± 25.7	4.6 ± 23.8	−1.2 ± 26.8
Δ-Triglycerides *	66.7 (58.3−75.2)	22.4 (−2.5–40.9)	−15.6 (−23.5–2.9)	27.5 (16.5–37.2)	25.0 (10.5–50.1)	23.9 (−3.4–55.5)	32.8 (15.8–45.2) ^1^	35.5 (−1.1–53.8) ^1^
Δ-HDL	−1.2 ± 15.4 ^a^	−0.6 ± 23.8 ^a^	−45.5 ± 55.8 ^b^	−12.0 ± 29.1 ^a^	−22.3 ± 30.7 ^1^	−24.7 ± 34.8 ^1^	−24.5 ± 28.0	−22.5 ± 31.9
Δ-LDL	14.7 ± 21.1	6.8 ± 38.0	4.5 ± 42.7	−10.6 ± 36.8	9.7 ± 30.2	0.9 ± 40.0	24.7 ± 145.6	−7.0 ± 45.7
Δ-Insulin *	46.8 (43.2–50.5)	70.8 (40.5–78.7)	44.3 (41.4–47.5)	32.6 (24.5–37.4)	58.3 (50.1–63.3) ^2^	58.8 (38.6–68.3)	53.4 (40.7–68.7) ^3^	58.8 (24.7–72.9) ^2^
Δ-HOMA-IR *	62.5 (47.5–77.6)	77.7 (54.9–84.9)	53.2 (49.0–55.4)	43.0 (34.5–47.8)	66.6 (56.3–73.1) ^a,1^	71.0 (50.1–77.1) ^b^	67.7 (52.6–79.1) ^a,b,3^	72.0 (51.7–78.5) ^a,b,2^
Δ-Leptin *	53.2 (50.8–55.6)	49.7 (49.2–70.8)	47.0 (40.3–59.6)	61.1 (56.5–64.1)	82.1 (73.3–88.2) ^1^	75.7 (65.9–81.5)	77.6 (71.0–85.0) ^2^	79.9 (62.3–83.8) ^1^
Δ-Adiponectin	−54.1 ± 25.6	−10.8 ± 43.9	−1.7 ± 39.4	−50.1 ± 58.6	−91.6 ± 92.2 ^a^	−81.2 ± 101.0 ^a^	−213.7 ± 193.9 ^b,1^	−105.1 ± 82.3 ^a^
Δ-CRP *	96.8 (95.9–97.8)	93.3 (85.9–96.2)	82.1 (78.7–88.9)	65.2 (54.9–80.9)	91.7 (80.7–96.1)	79.1 (50.4–87.8)	81.1 (69.4–91.3)	85.5 (54.4–95.4)
Δ-TG/HDL *	62.2 (50.1–74.2)	20.1 (−11.7–30.5)	1.9 (−27.5–15.0)	34.6 (21.7–47.5)	29.4 (7.7–58.3)	50.3 (−5.5–53.4)	48.3 (19.3–60.8)	46.2 (−9.6–58.1) ^1^
Δ-TC/HDL	17.5 ± 24.9 ^a,b^	9.9 ± 27.2 ^a,b^	24.8 ± 24.6 ^a^	3.1 ± 24.1 ^b^	22.8 ± 20.1	18.8 ± 20.1	21.5 ± 22.6	14.8 ± 20.8 ^1^
Δ-AIP	18.7 ± 39.0 ^a,b^	9.4 ± 73.4 ^b^	95.4 ± 175.0 ^a^	46.9 ± 58.7 ^a,b^	43.2 ± 77.0 ^1^	37.4 ± 150.0 ^1^	79.2 ± 121.2	64.2 ± 92.8

The results are given as the mean ± SD. * These results are given as median (interquartile range). BMI: Body mass index. SBP: Systolic blood pressure; DBP: Diastolic blood pressure; HOMA-IR: homeostasis model assessment of insulin resistance index. CRP: C-reactive protein. TG: triglycerides; TC: total cholesterol. AIP: atherogenic index of plasma. Different letters show significant differences between the means of the four groups of age: *p* < 0.05. Significant differences between patients with morbid obesity with %EWL <50 and those with %EWL ≥50% according to age: ^1^ *p* < 0.05; ^2^ *p* < 0.01 and ^3^ *p* < 0.001.

## References

[1] World Health Organization (2017). Obesity and Overweight. https://www.who.int/news-room/fact-sheets/detail/obesity-and-overweight.

[2] Sjöström L., Narbro K., Sjöström D., Karason K., Larsson B., Wedel H. (2007). Effects of bariatric surgery on mortality in swedish obese subjects. N. Engl. J. Med..

[3] Sjöström L. (2013). Review of the key results from the Swedish Obese Subjects (SOS) trial-a prospective controlled intervention study of bariatric surgery. J. Intern. Med..

[4] Still C.D., Wood G.C., Chu X., Manney C., Strodel W., Petrick A., Gabrielsen J., Mirshahi T., Argyropoulos G., Seiler J. (2014). Clinical factors associated with weight loss outcomes after Roux-en-Y gastric bypass surgery. Obesity.

[5] Camastra S., Muscelli E., Gastaldelli A., Holst J.J., Astiarraga B.D., Baldi S., Nannipieri M., Ciociaro D., Anselmino M., Mari A. (2013). Long-Term Effects of Bariatric Surgery on Meal Disposal and β-Cell Function in Diabetic and Nondiabetic Patients. Diabetes.

[6] MacLean L.D., Rhode B.M., Sampalis J., Forse R.A. (1993). Results of the surgical treatment of obesity. Am. J. Surg..

[7] Maggard M.A., Shugarman L.R., Suttorp M., Maglione M., Sugerman H.J., Livingston E.H., Nguyen N.T., Li Z., Mojica W.A., Hilton L. (2005). Meta-Analysis: Surgical Treatment of Obesity. Ann. Intern. Med..

[8] Sarzynski M., Jacobson P., Rankinen T., Carlsson B., Sjöström L., Bouchard C., Carlsson L.M.S. (2010). Associations of markers in 11 obesity candidate genes with maximal weight loss and weight regain in the SOS bariatric surgery cases. Int. J. Obes..

[9] Sugerman H.J., Londrey G.L., Kellum J.M., Wolf L., Liszka T., Engle K.M., Birkenhauer R., Starkey J.V. (1989). Weight loss with vertical banded gastroplasty and Roux-Y gastric bypass for morbid obesity with selective versus random assignment. Am. J. Surg..

[10] Stefanidis D., Kuwada T.S., Gersin K.S. (2010). The Importance of the Length of the Limbs for Gastric Bypass Patients—An Evidence-based Review. Obes. Surg..

[11] Nguyen N.T., Rivers R., Wolfe B.M. (2003). Factors associated with operative outcomes in laparoscopic gastric bypass. J. Am. Coll. Surg..

[12] Melton G.B., Steele K.E., Schweitzer M.A., Lidor A.O., Magnuson T.H. (2007). Suboptimal Weight Loss after Gastric Bypass Surgery: Correlation of Demographics, Comorbidities, and Insurance Status with Outcomes. J. Gastrointest. Surg..

[13] Sans A., Bailly L., Anty R., Sielezenef I., Gugenheim J., Tran A., Gual P., Iannelli A. (2017). Baseline Anthropometric and Metabolic Parameters Correlate with Weight Loss in Women 1-Year After Laparoscopic Roux-En-Y Gastric Bypass. Obes. Surg..

[14] Campos G.M. (2008). Factors Associated With Weight Loss After Gastric Bypass. Arch. Surg..

[15] Coupaye M., Sabate J.M., Castel B., Jouët P., Clerici C., Msika S., LeDoux S. (2010). Predictive Factors of Weight Loss 1 Year after Laparoscopic Gastric Bypass in Obese Patients. Obes. Surg..

[16] Barhouch A.S., Padoin A.V., Casagrande D.S., Chatkin R., Süssenbach S.P., Pufal M.A., Rossoni C., Mottin C.C. (2015). Predictors of Excess Weight Loss in Obese Patients After Gastric Bypass: A 60-Month Follow-up. Obes. Surg..

[17] Ma Y., Pagoto S.L., Olendzki B.C., Hafner A.R., Perugini R.A., Mason R., Kelly J.J. (2006). Predictors of Weight Status following Laparoscopic Gastric Bypass. Obes. Surg..

[18] Contreras J.E., Santander C., Court I., Bravo J. (2013). Correlation Between Age and Weight Loss after Bariatric Surgery. Obes. Surg..

[19] Garrido-Sanchez L., Murri M., Rivas-Becerra J., Ocaña-Wilhelmi L., Cohen R., Garcia-Fuentes E., Tinahones F. (2012). Bypass of the duodenum improves insulin resistance much more rapidly than sleeve gastrectomy. Surg. Obes. Relat. Dis..

[20] García-Fuentes E., Garrido-Sanchez L., Garcia-Almeida J.M., Garcia-Arnes J., Gallego-Perales J.L., Rivas-Marin J., Morcillo S., Cardona I., Soriguer F. (2008). Different Effect of Laparoscopic Roux-en-Y Gastric Bypass and Open Biliopancreatic Diversion of Scopinaro on Serum PYY and Ghrelin Levels. Obes. Surg..

[21] De Hollanda A., Ruiz T., Jiménez A., Flores L., Lacy A., Vidal J. (2014). Patterns of Weight Loss Response Following Gastric Bypass and Sleeve Gastrectomy. Obes. Surg..

[22] Reinhold R.B. (1982). Critical analysis of long term weight loss following gastric bypass. Surg. Gynecol. Obstet..

[23] Christou N.V., Look D., MacLean L.D. (2006). Weight Gain After Short- and Long-Limb Gastric Bypass in Patients Followed for Longer Than 10 Years. Ann. Surg..

[24] Shen S.-W., Lu Y., Li F., Yang C.-J., Feng Y.-B., Li H.-W., Yao W.-F., Shen Z.-H. (2018). Atherogenic index of plasma is an effective index for estimating abdominal obesity. Lipids Health Dis..

[25] Lemieux I., Lamarche B., Couillard C., Pascot A., Cantin B., Bergeron J., Dagenais G.R., Després J.-P. (2001). Total Cholesterol/HDL Cholesterol Ratio vs LDL Cholesterol/HDL Cholesterol Ratio as Indices of Ischemic Heart Disease Risk in Men. Arch. Intern. Med..

[26] Statements P. (2012). Standards of medical care in diabetes—2012. Diabetes Care.

[27] Chobanian A.V., Bakris G.L., Black H.R., Cushman W.C., Green L.A., Izzo J.L., Jones D.W., Materson B.J., Oparil S., Wright J.T. (2003). The Seventh Report of the Joint National Committee on Prevention, Detection, Evaluation, and Treatment of High Blood Pressure: The JNC 7 Report. JAMA.

[28] Brethauer S.A., Kim J., Chaar M.E., Papasavas P., Eisenberg D., Rogers A. (2015). Standardized outcomes reporting in metabolic and bariatric surgery. Obes. Surg..

[29] Villarreal-Calderón J.R., Cuéllar R.X., Ramos-González M.R., Rubio-Infante N., Castillo E.C., Elizondo-Montemayor L. (2019). Interplay between the Adaptive Immune System and Insulin Resistance in Weight Loss Induced by Bariatric Surgery. Oxid. Med. Cell Longev..

[30] Cottam D.R., Mattar S.G., Barinas-Mitchell E., Eid G., Kuller L., Kelley D.E., Schauer P.R. (2004). The Chronic Inflammatory Hypothesis for the Morbidity Associated with Morbid Obesity: Implications and Effects of Weight Loss. Obes. Surg..

[31] Leggio M., Lombardi M., Caldarone E., Severi P., D’Emidio S., Armeni M., Bravi V., Bendini M.G., Mazza A. (2017). The relationship between obesity and hypertension: An updated comprehensive overview on vicious twins. Hypertens. Res..

[32] Park C.H., Nam S.-J., Choi H.S., Kim K.O., Kim D.H., Kim J.-W., Sohn W., Yoon J.H., Jung S.H., Korean Research Group for Endoscopic Management of Metabolic Disorder and Obesity (2019). Comparative Efficacy of Bariatric Surgery in the Treatment of Morbid Obesity and Diabetes Mellitus: A Systematic Review and Network Meta-Analysis. Obes. Surg..

[33] Liu F., He J., Wang H., Zhu D., Bi Y. (2020). Adipose Morphology: A Critical Factor in Regulation of Human Metabolic Diseases and Adipose Tissue Dysfunction. Obes. Surg..

[34] Garrido-Sánchez L., Tomé M., Santiago-Fernández C., García-Serrano S., García-Fuentes E., Tinahones F.J. (2017). Adipose tissue biomarkers involved in early resolution of type 2 diabetes after bariatric surgery. Surg. Obes. Relat. Dis..

[35] Diamantis T., Apostolou K.G., Alexandrou A., Griniatsos J., Felekouras E., Tsigris C. (2014). Review of long-term weight loss results after laparoscopic sleeve gastrectomy. Surg. Obes. Relat. Dis..

[36] Yu J., Zhou X., Li L., Li S., Tan J., Li Y., Sun X. (2014). The Long-Term Effects of Bariatric Surgery for Type 2 Diabetes: Systematic Review and Meta-analysis of Randomized and Non-randomized Evidence. Obes. Surg..

[37] Printen K.J., Mason E.E. (1977). Gastric bypass for morbid obesity in patients more than fifty years of age. Surg. Gynecol. Obstet..

[38] Livingston E.H., Huerta S., Arthur D., Lee S., De Shields S., Heber D. (2002). Male Gender is a Predictor of Morbidity and Age a Predictor of Mortality for Patients Undergoing Gastric Bypass Surgery. Ann. Surg..

[39] Dobiášová M. (2004). Atherogenic Index of Plasma [Log(Triglycerides/HDL-Cholesterol)]: Theoretical and Practical Implications. Clin. Chem..

[40] Murri M., García-Fuentes E., García-Almeida J.M., Garrido-Sanchez L., Mayas M.D., Bernal R., Tinahones F.J. (2009). Changes in Oxidative Stress and Insulin Resistance in Morbidly Obese Patients After Bariatric Surgery. Obes. Surg..

[41] Piché M.-È., Martin J., Cianflone K., Bastien M., Marceau S., Biron S., Hould F.-S., Poirier P. (2014). Changes in predicted cardiovascular disease risk after biliopancreatic diversion surgery in severely obese patients. Metabolism.

[42] Perathoner A., Weißenbacher A., Sucher R., Laimer E., Pratschke J., Mittermair R. (2013). Significant Weight Loss and Rapid Resolution of Diabetes and Dyslipidemia During Short-Term Follow-Up After Laparoscopic Sleeve Gastrectomy. Obes. Surg..

[43] Khaitan L., Shea B.J. (2020). Laparoscopic vertical sleeve gastrectomy, long and short-term impact on weight loss and associated co-morbidities. Ann. Transl. Med..

[44] Bhandari M., Reddy M., Kosta S., Mathur W., Fobi M., Mohit B., Manoj R., Susmit K., Winni M., Mathias F. (2019). Laparoscopic sleeve gastrectomy versus laparoscopic gastric bypass: A retrospective cohort study. Int. J. Surg..

[45] Osland E., Yunus R.M., Khan S., Memon B., Memon M.A. (2017). Weight Loss Outcomes in Laparoscopic Vertical Sleeve Gastrectomy (LVSG) Versus Laparoscopic Roux-en-Y Gastric Bypass (LRYGB) Procedures. Surg. Laparosc. Endosc. Percutaneous Tech..

